# Relationship between bedtime, nighttime sleep duration, and anxiety symptoms in preschoolers in China

**DOI:** 10.3389/fpsyg.2024.1290310

**Published:** 2024-01-17

**Authors:** Shuang-Yan Qiu, Xiao-Na Yin, Yanni Yang, Ting Li, Dali Lu, Jie-Min Li, Wei-Kang Yang, Guo-Ming Wen, Jing-Yu Zhang, Yan Zhang, Hai-Yan Lei, Xin Wang, Jian-Bo Wu

**Affiliations:** ^1^Shenzhen Longhua Maternity and Child Healthcare Hospital, Shenzhen, China; ^2^Xinhe Experimental School, Shenzhen, China; ^3^Key Laboratory of Brain, Cognition and Education Sciences, Ministry of Education, Institute for Brain Research and Rehabilitation, Guangdong Key Laboratory of Mental Health and Cognitive Science, South China Normal University, Guangzhou, China

**Keywords:** bedtime, nighttime sleep duration, preschoolers, anxiety symptoms, boundary

## Abstract

**Background:**

Sleep problems in preschoolers are becoming increasingly prominent, and the association between sleep status and anxiety symptoms has attracted growing attention. However, studies investigating the relationship between bedtime and nighttime sleep duration in preschoolers and their anxiety symptoms remain scant. We used the large sample data from the Longhua Cohort Study of Children in Shenzhen, China (LCCS) to analyze the association between bedtime and sleep in preschoolers and their anxiety symptoms.

**Methods:**

A cross-sectional study of 69,138 preschoolers in Longhua District, Shenzhen, China was conducted in 2022. Data on sociodemographic characteristics of families, bedtime, nighttime sleep duration of preschoolers, and their anxiety symptoms (measured by the Spence Preschool Children Anxiety Scale) were collected through a structured questionnaire completed by the parents. Using binary logistic regression models, the relationship between bedtime, nighttime sleep duration, and childhood anxiety symptoms was examined.

**Results:**

The bedtimes of preschoolers were concentrated between 21:01–22:00 (52.41%). Among the preschoolers, 38.70% had bedtimes later than 22:00, and 75.49% had insufficient nighttime sleep duration. The positive screening rate for anxiety symptoms among preschoolers was 3.50%. After adjusting for confounding factors using binary logistic regression models, compared with preschoolers with bedtime ≤21:00, The OR (95%CI) values of anxiety in preschoolers with bedtime ≥23:01, 22:01–23:00 and 21:01–22:00 were 2.86 (2.21–3.69), 1.51 (1.27–1.79) and 1.48 (1.26–1.76), respectively. Compared with those with sufficient nighttime sleep duration, the OR (95%CI) of children with nighttime sleep duration less than 9 h was 1.36 (1.23–1.51).

**Conclusion:**

An association exists between bedtime and nighttime sleep duration in preschoolers and their anxiety symptoms. Preschoolers with 21:00 for bedtime and a nighttime sleep duration of 10 h may have lower anxiety symptoms. These findings support the importance of adequate sleep for preventing anxiety symptoms in children.

## Introduction

Anxiety is an inner uneasiness or unwarranted fear that lacks an obvious objective cause (Overgaard et al., 2014). Over the past 30 years, research efforts in adult and adolescence anxiety disorders have increased our understanding of anxiety. However, we know relatively little about the anxiety symptoms in preschoolers (Wang and Zhao, 2015). Research suggests that anxiety symptoms have an early age of onset (Rapee et al., 2009). A US study revealed that approximately 9.5% of preschoolers have anxiety symptoms (Egger and Angold, 2006). Approximately 10% of preschoolers in China have anxiety symptoms (Zhi-Wei et al., 2013; Jin-Lian et al., 2014). Anxiety symptoms in preschoolers can adversely affect children’s development (Paulus et al., 2015; Morgan et al., 2019). Without timely intervention, anxiety symptoms in preschoolers may continue into adulthood (Wichstrøm et al., 2013). Globally, anxiety-related productivity loss is estimated at $1 trillion per year (Chisholm et al., 2016). Therefore, it is worthwhile to study the risk factors of anxiety symptoms in preschoolers, which is helpful for the prevention of anxiety symptoms in preschoolers.

Sleep deprivation has been shown to reduce the occurrence of positive emotions and to alter the way individuals understand, express, and modify these emotions, potentially triggering anxious emotions. Sleep plays a key role in mental health and psychosocial adjustment across the lifespan, especially in children (Palmer and Alfano, 2017). At present, children’s sleep problems are common and have become a global public health problem. The incidence of sleep problems in preschool children is 20–45% (Fricke-Oerkermann et al., 2007; Simola et al., 2010). A cross-cultural study of many countries and regions showed that the incidence of sleep problems in Chinese children was significantly higher than that in European and American countries (Mindell et al., 2010). Sleep problems in preschoolers manifest mainly as short nighttime sleep duration and late bedtime (Mindell et al., 2013; Asarnow et al., 2014), which can negatively affect their quality of life (Ekinci et al., 2016) and cause long-term sleep problems (Hatzinger et al., 2013). Sleep is an important part of human life activities, and the sleep time of people accounts for about one third of their life. As a basic brain activity during the early growth and development of children, sleep has an important impact on their mental health and emotional regulation.

The association between sleep and anxiety symptoms in preschoolers is worth studying, which can provide scientific basis for early identification and intervention of anxiety symptoms and promote their healthy growth. At present some research suggests that about the adolescents and adult of the relationship between sleep and anxiety symptoms may also operate in both directions (Kahn et al., 2013). Individuals suffering from anxiety symptoms often experience sleep deprivation (Johnson et al., 2006). Sleep deprivation can trigger or further exacerbate anxiety symptoms (Seo et al., 2020). However, previous studies have primarily focused on sleep patterns in adolescents, and few studies on preschoolers have been conducted (Chellappa and Aeschbach, 2021). At present, the sleep problems of preschoolers are becoming more and more serious, but there are few studies on children’s sleep and anxiety symptoms. In addition, existing studies on the association between sleep and anxiety symptoms in preschoolers employed small sample sizes (Covington et al., 2019). This study used the large sample data from the Longhua Children Cohort Study in Shenzhen, China (LCCS) to analyze the association between bedtime, nighttime sleep duration, and anxiety symptoms in preschoolers.

## Methods

### Study population

The participants were recruited from the 2022 LCCS survey. The LCCS is an ongoing prospective cohort study of preschoolers in the Longhua District, Shenzhen, China. In 2022, a questionnaire survey was distributed among 250 kindergarten parents in Longhua District, Shenzhen, China. Exclusion criteria included children with severe physical illnesses or mental disorders. A total of 75,557 questionnaires were distributed, and 71,069 questionnaires were returned, with a recovery rate of 94.06%. The final sample size was 69,138 (excluding 1,931 questionnaires with incomplete information). This study was approved by the Ethics Committee of Shenzhen Longhua Maternal and Child Health Hospital (ethics license number: 2016102501) and the School of Public Health, Sun Yat-sen University (ethics license number: 2015–016). Informed consent was obtained from all the study participants.

### Data collection

Kindergarten principals and physicians were trained in the use of questionnaires. The invitations were extended to the parents of young children to participate. After obtaining parental informed consent, a questionnaire was administered. An electronic questionnaire was employed for this survey. Information on the overnight bedtime and nighttime sleep duration of the preschoolers, anxiety status, general information about the children, and sociodemographic characteristics of the parents were collected using a questionnaire.

### Measurement of bedtime and nighttime sleep duration in preschoolers (major exposure variables)

Parents of the children were surveyed using a questionnaire. This study investigated bedtime and nighttime sleep duration in preschoolers (Table 1). Bedtime = (bedtime from Monday to Friday * 5 + Saturday and Sunday bedtime * 2) / 7, using the classification of other literature (Cao et al., 2018), bedtime was divided into four categories: 21:00, 21:01–22:00, 22:01–23:00, 23:01. Nighttime sleep duration = (Monday to Friday nighttime sleep duration * 5 + Saturday and Sunday nighttime sleep duration * 2) / 7, According to the American Association of Sleep Medicine guidelines (Berry et al., 2012) and previous studies (Mindell et al., 2009). Nighttime sleep duration was divided into <9 h, 9–10 h, and 10 h; 10 h was defined as adequate sleep.

**Table 1:** Sleep status survey of preschoolers.

No	Questions
Q1.1	During the past month, what is your child’s usual bedtime from Monday to Friday?
Q1.2	During the past month, what is your child’s usual weekend bedtime?
Q2.1	During the past month, how much is your child nighttime sleep duration per day from Monday to Friday?
Q2.2	During the past month, how much is your child nighttime sleep duration per day on weekends?

### Measurement of anxiety in preschoolers (outcome variable)

Anxiety symptoms were measured using the Chinese version of the Spence Preschool Children Anxiety Scale (SPAS). The SPAS Chinese version was specifically designed by Chinese researchers to measure anxiety symptoms in preschoolers aged 3–6 years. The scale has good reliability and validity, with a Cronbach’s α coefficient of 0.95 (Wang and Zhao, 2015). The scale has a clear factor structure, good internal consistency and reasonable criterion validity. There is a strong correlation between the scale and other anxiety symptom scales. The 5-point Likert scoring method was used, with “never” = 0, “rarely” = 1, “sometimes” = 2, “often” = 3, and “always” = 4. The total score of each dimension was obtained by adding the scores of each dimension and all dimensions to obtain the total anxiety score ranging from 0 to 112. Higher scores indicate serious anxiety symptoms. A total score of 48 was defined as positive screening for anxiety symptoms (Hu et al., 2021).

### Covariates

The following confounding covariates were included in the analysis: sex, number of children in the family, maternal and paternal educational levels, monthly household income, and parental marital status.

### Statistical analysis

Here, we describe bedtime and nighttime sleep duration in preschoolers. Chi-square tests were used to compare the sociodemographic characteristics of children who screened positive for anxiety symptoms. The associations of bedtime and nighttime sleep duration with anxiety symptoms in preschoolers were analyzed using a binary logistic regression model. The results are presented as ORs with 95% CI. Statistical significance was set at a two-tailed test with *p* < 0.05. Data management and statistical analyses were performed using Statistical Package for the Social Sciences (version 25.0; SPSS Inc., Chicago, IL, USA).

## Results

### Social characteristics and anxiety symptoms in preschoolers

Table 2 presents the participants’ sociodemographic characteristics. Of the 69,138 children enrolled in the study, 2,399 (3.50%) demonstrated anxiety symptoms. Low parental education level, low family income, having only one child, and single-parent families were risk factors for anxiety symptoms (Table 2).

**Table 2:** Social characteristics and anxiety symptoms in preschoolers.

Characteristics	Anxiety symptoms		χ^2^/F	*p*-value
	No (*N* = 66,739, 96.50%)	Yes (*N* = 2,399, 3.50%)		
Child’s age [mean ± SD (years)]	4.90 ± 0.90	4.80 ± 0.90	0.13	0.71
Gender [*n* (%)]			18.02	<0.001
Male	35,681 (96.80)	1,177 (3.20)		
Female	31,058 (96.21)	1,222 (3.79)		
Single child status [*n* (%)]			4.88	<0.05
Yes	45,420 (96.42)	1,684 (3.58)		
No	21,319 (96.75)	715 (3.24)		
Maternal education level [*n* (%)]			153.81	<0.001
Junior high school or lower	9,654 (94.56)	550 (5.44)		
High school	13,767 (96.33)	524 (3.67)		
College	41,177 (97.01)	1,265 (2.99)		
Master’s degree or above	2,231 (97.38)	60 (2.62)		
Paternal education level [*n* (%)]			88.04	<0.001
Junior high school or lower	8,873 (95.19)	448 (4.81)		
High school	13,944 (96.03)	576 (3.96)		
College	40,552 (96.92)	1,285 (3.08)		
Master’s degree or above	3,370 (97.40)	90 (2.60)		
Monthly household income [*n* (%)]			183.51	<0.001
<¥10,000	10,868 (94.53)	628 (5.47)		
¥10,000–20,000	22,751 (96.53)	817 (3.47)		
¥20,000–30,000	14,231 (97.06)	430 (2.94)		
¥30,000–40,000	7,817 (97.23)	222 (2.77)		
>¥40,000	11,072 (97.34)	302 (2.66)		
Family marriage status			9.89	<0.001
Married	63,682 (96.56)	2,268 (3.44)		
Unmarried/Divorced	957 (97.05)	29 (2.95)		
Widowed/Remarried	2,100 (95.36)	102 (4.64)		

Means (SD) are presented for continuous variables, and N (%) is presented for non-continuous variables. χ^2^, chi-square test. F, F variance analysis. SD, standard deviation. N (%), quantity (proportion).

### Distribution of bedtime and nighttime sleep duration in preschoolers

The bedtime order for preschoolers was 21:01–22:00 (52.41%), 22:01–23:00 (36.38%), 21:00 (8.89%), and 23:01 (2.32%). In total, 38.70% of the children had a bedtime later than 22:00, the mean nighttime sleep duration was 9.23 h ± 1.03 h, and 75.49% of children were sleep deprived (see Table 3). The proportion of boys with bedtime ≤21:00 or nighttime sleep duration ≥10 h was slightly higher than that of girls (*p* < 0.05).

**Table 3:** Distribution of bedtime and nighttime sleep duration in preschoolers.

Characteristics	Sum	Sex		χ^2^/F	*p*-value
		Male (*N* = 36,858, 53.30%)	Female (*N* = 32,280, 46.70%)		
Bedtime					
≤21:00	6,149 (8.89)	3,318 (9.0)	2,831 (8.8)	10.64	<0.05
21:01–22:00	36,233 (52.41)	19,483 (52.9)	16,750 (51.9)		
22:01–23:00	25,151 (36.38)	13,208 (35.8)	11,943 (37.0)		
≥23:00	1,605 (2.32)	849 (2.3)	756 (2.3)		
Nighttime sleep duration					
≥10 h	16,945 (24.51)	8,856 (35.4)	8,089 (34.0)	18.22	<0.001
9–10 h	28,169 (40.74)	14,949 (40.6)	13,220 (41.0)		
<9 h	24,024 (34.75)	13,053 (24.0)	10,971 (25.0)		

### Binary logistic regression analysis of bedtime and nighttime sleep duration with anxiety symptoms (primary outcome)

After adjusting for confounding factors using binary logistic regression models, compared with preschoolers with bedtime ≤21:00, The OR (95%CI) values of anxiety in preschoolers with bedtime ≥23:01, 22:01–23:00 and 21:01–22:00 were 2.86 (2.21–3.69), 1.51 (1.27–1.79) and 1.48 (1.26–1.76), respectively. Compared with those with sufficient nighttime sleep duration, the OR (95%CI) of children with nighttime sleep duration less than 9 h was 1.36 (1.23–1.51) (see Table 4).

**Table 4:** Binary logistic regression analysis of bedtime and nighttime sleep duration with anxiety symptoms.

Sleep condition	No. of children	Cases (*n*%)	AOR (95% CI)
Bedtime			
≤21:00	6,149	163 (2.65)	
21:01–22:00	36,233	1,296 (3.57)	1.48 (1.26–1.76) ***
22:01–23:00	25,151	839 (3.33)	1.51 (1.27–1.79) ***
≥23:00	1,605	101 (6.29)	2.86 (2.21–3.69) ***
Nighttime sleep duration			
≥10 h	24,024	788 (3.28)	
9–10 h	28,169	865 (3.07)	0.95 (0.86–1.05)
<9 h	16,945	746 (4.40)	1.36 (1.23–1.51) ***

AOR, Adjusted odds ratio. OR adjusted for child’s sex, number of children in the family, maternal and paternal education levels, monthly household income, and parental marital status. CI, Confidence interval. Ref, Reference. ***p < 0.001.

## Discussion

### Preschoolers have experienced prominent sleep problems

Only 8.89% of local preschoolers sleep by 21:00; 75.49% of children sleep for less than 10 h, and the average nighttime sleep duration is 9.23 ± 1.03 h. The above data indicate that preschool children in this area have late bedtimes and lack of nighttime sleep duration. This finding is consistent with the average bedtime and nighttime sleep durations of preschoolers in 10 cities in China (Yangyang and Nan, 2020). Some previous studies have found that preschoolers in Asia sleep less than those in the United States. A previous survey revealed that the average bedtime for preschoolers in other Asian countries was between 21:41 and 21:55, and their nighttime sleep duration was between 9.25 and 9.25 h (Komada et al., 2011), respectively. However, in the United States, the average bedtime of preschoolers is 20:39, and the average sleep time is 10.47 h (Scharf et al., 2013), indicating that preschoolers have a early bedtime and adequate sleep duration. Some studies have shown that no gender differences exist in the sleep status of preschoolers (Owens et al., 2000). Our study also found that boys sleep earlier and longer than girls.

This may be due to the increased activity of boys, leading to deeper nighttime sleep.

### Late night bedtime and inadequate sustained sleep duration were associated with anxiety symptoms in preschoolers

This study uncovered an association between nighttime bedtime, sleep duration, and anxiety symptoms in preschoolers. We found preschoolers with 21:00 for bedtime and a nighttime sleep duration of 10 h may have lower anxiety symptoms. The boundary values can give parents as a reference. Human sleep has similarities to sleep in other mammals. For instance, when mice were anxious, they tended to spend more time in the dark; nonetheless, tests revealed that after sufficient sleep, they spent more time in the light than searching for darkness, indicating that adequate sleep reduced anxiety levels (Yu et al., 2022).

In terms of neurotransmitters, late bedtime mainly affects the circadian rhythm of children’s sleep, whereas the sleep duration mainly affects sleep length and depth (Akacem et al., 2016). The sleep circadian rhythm directly affects the release of endocrine hormones. Late bedtime leads to an abnormal release of hormones, which can lead to emotional problems in children (Zohar et al., 2005; Wu et al., 2022). Another study examined the relationship between sleep and psychological symptoms using a crossover lag model and found that sustained reductions in sleep duration and decreased sleep quality predicted higher levels of anxiety symptoms (Kelly and El-Sheikh, 2014), which may be because insufficient sleep induces anxious mood in the amygdala and prefrontal skin. Late nighttime bedtime and insufficient sleep duration increased the risk of anxiety symptoms (Suveg and Zeman, 2004).

From a psychological perspective, emotional evaluation is an important part of the emotional generation process and can minimize the negative evaluation of stimuli, thereby reducing anxiety symptoms (Gross, 2014; Werner et al., 2015). Early bedtime and continuous sleep can help improve anxiety symptoms in preschoolers (Covington et al., 2019). Studies have to sleep restriction of people of different ages, found that sleep more negative effects on the child’s emotions (Motomura et al., 2013). In addition, sleep deprivation can significantly alter an individual’s positive behaviors, increasing the likelihood that they are unhappy (Libedinsky et al., 2013).

The main strength of our study is that we analyzed the association between sleep status and anxiety symptoms in preschoolers through large-sample data and discovered that preschoolers with a bedtime of 21:00 and a sleep duration of 10 h may have lower anxiety symptoms. However, this study has the following limitations. First, in investigating the sleep status in children, we used a questionnaire for retrospective investigation and may have encountered recall bias. Second, only two factors were considered in this study: late bedtime and sleep duration. Sleep conditions, including nap habits, difficulty sleeping, night waking, nightmares, and other factors (Thorpe et al., 2015), were not accounted for in this study and thus must be considered in future investigations. Third, our study revealed that bedtime and nighttime sleep duration were associated with anxiety symptoms in preschoolers, but did not prove that sleep patterns directly caused anxiety symptoms in preschoolers. Fourth, because cultural and environmental factors may significantly influence sleep habits, we should be more cautious about generalizing our results from one region to another.

## Conclusion

An association exists between bedtime and nighttime sleep duration in preschoolers and their anxiety symptoms. Preschoolers with 21:00 for bedtime and a nighttime sleep duration of 10 h may have lower anxiety symptoms. These findings support the importance of adequate sleep for preventing anxiety symptoms in children.

## Data availability statement

The original contributions presented in the study are included in the article/supplementary material, further inquiries can be directed to the corresponding author.

## Ethics statement

This study was approved by the Ethics Committee of Shenzhen Longhua Maternal and Child Health Hospital (ethics license number: 2016102501) and the School of Public Health, Sun Yat-sen University (ethics license number: 2015-016). Informed consent was obtained from all the study participants.

## Author contributions

S-YQ: Conceptualization, Data curation, Investigation, Writing – original draft, Funding acquisition. X-NY: Data curation, Funding acquisition, Methodology, Writing – original draft. YY: Data curation, Project administration, Supervision, Writing – review & editing. TL: Methodology, Writing – review & editing. DL: Data curation, Methodology, Writing – review & editing. J-ML: Validation, Visualization, Writing – review & editing. W-KY: Software, Supervision, Writing – original draft. G-MW: Methodology, Supervision, Writing – original draft. J-YZ: Methodology, Project administration, Writing – original draft. YZ: Methodology, Project administration, Writing – original draft. H-YL: Data curation, Investigation, Writing – original draft. XW: Data curation, Formal analysis, Investigation, Methodology, Resources, Validation, Writing – original draft. J-BW: Conceptualization, Data curation, Formal analysis, Funding acquisition, Investigation, Methodology, Project administration, Resources, Software, Supervision, Validation, Visualization, Writing – original draft, Writing – review & editing.
